# Protective effects of vitamins/antioxidants on occupational noise‐induced hearing loss: A systematic review

**DOI:** 10.1002/1348-9585.12217

**Published:** 2021-03-31

**Authors:** Milad Abbasi, Behnaz Pourrajab, Mohammad Osman Tokhi

**Affiliations:** ^1^ Social Determinants of Health Research Center Saveh University of Medical Sciences Saveh Iran; ^2^ Department of Nutrition School of Public Health Iran University of Medical Sciences Teheran Iran; ^3^ School of Engineering London South Bank London UK

**Keywords:** antioxidants, NIHL, occupational noise‐induced hearing loss, vitamins

## Abstract

**Objectives:**

Occupational noise‐induced hearing loss (NIHL) due to industrial, military, and other job ‐related noise exposure can cause harmful health issues to occupied workers, but may also be potentially preventable. Vitamins/antioxidant have been studied as therapeutic strategies to prevent and/or delay the risks of human diseases as well as NIHL .So, this study was conducted to systematically review the protective effects of vitamins/antioxidants on occupational NIHL.

**Methods:**

Online databases including PubMed/Medline, Scopus, Web of Science, EMBASE, Science Direct, and Google Scholar were systematically searched up to 12 January 2021. Based on 6336 potentially relevant records identified through the initial search in the databases, 12 full‐text publications were retrieved, one of which can be viewed as two separate trials, because it has studied the effects of two different antioxidants (ginseng and NAC) on NIHL, separately.

**Results:**

A review of the studies shows that vitamin B12, folic acid, and N‐acetylcysteine (NAC) have a considerable protective effect on NIHL. However, these protective effects are not yet specified in different frequencies. The findings regarding the protective effects of other antioxidants are inconsistent in this field.

**Conclusion:**

Vitamin B12, folic acid, and NAC may have a protective effect as an antioxidant on reducing occupational hearing loss. For a conclusive evidence of vitamin/antioxidant protective therapies, future studies with precise criteria for noise exposure and similar outcome parameters are required.

**Highlight**

1. Vitamin B12, folic acid, and NAC have a considerable protective effect on NIHL.
2. Findings regarding the protective effect of vitamins E, C, and A on NIHL are inconsistent.
3. Future studies with precise criteria for noise exposure and similar outcome parameters are required.

## 1 INTRODUCTION

Workers in most industries are exposed to noise pollution. Noise as an unwanted sound can cause adverse health effects and safety risks to occupied workers. The most recognized effects on the human health caused by this harmful agent are annoyance and perception,^1^, ^2^ cardiovascular diseases and hypertension,^3^, ^4^ hearing loss,^5^ sleep disorders,^6^ stress,^7^ psychological effects,^8^ well‐being disorders and satisfaction,^9^, ^10^ and cognitive performance defect.^11^ Noise‐induced hearing loss (NIHL) is known as more significant effect among the others, so it has attracted researchers' attention.

NIHL refers to hearing loss caused by prolonged exposure to high levels of noise in the workplace and is categorized as a substantial occupational disease. There are two types of hearing loss, known as temporary threshold shift (TTS) and permanent threshold shift (PTS).^12^, ^13^ TTS is typically caused by traumatizing stimulus spectrum, which is affected by the level and duration of exposure. It is also usually caused by acute noise exposure and can be reversible in a few days depending on the exposure level and duration.^14^ PTS is the same as TTS, except that it is an irreversible change of hearing threshold.^13^ In addition to noise, there are some other factors that can cause TTS and PTS. NIHL has a prevalence rate of 16%, which varies from 7% of the population in industrialized countries to 21% in developing countries.^5^, ^15^ Neitzel et al^16^ have reported that the prevalence rate of hearing loss among the United States working population is 13%. It is also estimated that if NIHL is reduced by 20%, the financial benefit of $58 billion to $152 billion annually would be reached.^16^ In addition to the direct cost, hearing loss has significant effects on different aspects of daily living of affected people. It is documented that hearing loss has an adverse effect on cognitive performance, quality of life and work, physical well‐being, peers and social support, social relationships, motor skills, and psychological aspects.^17^, ^18^ Based on the aforementioned issues, using effective strategies for preventing NIHL, such as reduced noise exposure through engineering and administrative control measures, training interventions, providing hearing protection devices, and vitamin/antioxidant intake, is substantial.^19^, ^20^, ^21^ Engineering and administrative control measures require a lot of funding for implementation and maintenance, therefore sometimes it is not cost‐effective. Also, providing hearing protective devices, in addition to imposing a large financial burden, may not be effective due to improper use and interference with routine work.^22^

Since oxidative stress plays an important role in hearing loss, it can be expected that the consumption of antioxidants will create a barrier to NIHL.^23^ In fact, it is noted that high levels of noise are likely to result in damaging free radicals, and some studies have shown that during and after noise exposure, reactive oxygen species (ROS), reactive nitrogen species (RNS), and lipid peroxides all increase, leading to hearing loss.^24^, ^25^ Although the exact mechanism is not yet clear, some pathways have been suggested. Noise affects the cochlea metabolically and mechanically at some points, leading to several types of damage. At the level of hair cells, noise can lead to overdriving of mitochondria, toxicity stimulation in the connections between inner hair cells and auditory nerve fibers, and ischemia/reperfusion effects on the cochlea's blood source. Each of these can lead to increase in ROS, which can damage DNA and the cell membrane and act as a starting factor for apoptosis. The final consequence is hair cell lesion and loss of hearing from a combination of necrosis and apoptosis.^24^ On the other hand, the primary action of antioxidants is to reduce the damaging effects of oxygen on biomolecules. Many antioxidant mechanisms exist but primarily include the scavenging or blocking of free radicals.^26^ Different types of free radicals are formed and each antioxidant has a distinct efficacy.^27^ Some review studies have examined the association between hearing loss/sensorineural hearing loss/age‐related hearing loss and vitamins/antioxidants.^28^, ^29^, ^30^ However, according to our search results, so far no study has been done to systematically review the relationship between vitamins/antioxidants on occupational NIHL. Only, Alvarado et al in their review on a small number of studies showed that combination of some or all of the antioxidants, such as NAC, vitamins A, C, E, and magnesium (Mg), can produce synergism and/or redundancy in their mechanisms of action, potentiating the positive effect over noise overexposure, and also considered these otoprotective agents as a hopeful new therapeutic strategy for ameliorating, delaying, or even preventing the impact of noise on hearing.^31^ So, the current study is conducted to systematically review the protective effects of vitamins/antioxidants on occupational NIHL.

## 2 METHOD

The current systematic review is written by referring to the Preferred Reporting Items for Systematic Reviews and Meta‐Analyses (PRISMA) guidelines.^32^

### 2.1 Search strategy

Online databases including PubMed/Medline, Scopus, Web of Science, EMBASE, Science Direct, and Google Scholar were systematically searched up to 12 January 2021 to find related articles published to this date, without language, time, or any other limitations. There was no specific limitation for the type of studies. The following keywords were used to construct the search strategy: ((micronutrient*) OR (vitamin*) OR (antioxidant*) OR ("Nutritional supplement*") OR ("dietary supplement*") OR (N‐Acetylcysteine) OR (Acetylcysteine) OR (NAC)) AND (("Hearing Disorders") OR ("Distorted Hearing") OR ("Acoustic Trauma") OR ("noise induced hearing loss") OR (NIHL) OR ("hearing damage") OR ("occupational hearing loss") OR ("noise injury") OR (noise exposure) OR ("occupational deafness")).

### 2.2 Eligibility criteria

All published studies (interventional and observational studies) that have reported the relation between vitamins/antioxidants with occupational NIHL in adult individuals (over 18 years old) were included for further consideration. Studies in which subjects were exposed to noise and their serum levels of vitamins/antioxidants or the effects of taking vitamins/antioxidants that have been studied and appeared to be applicable to people who are exposed to occupational noise also meet criteria for this review. Language restriction and specific time frame were not applied for search and all studies published on this topic up to January 2021 were reviewed.

Exclusion criteria in this study were:

- Animal and in vitro studies
- Studies on the effects of vitamins/antioxidants on hearing loss caused by use of drug, accidents, illness, music, and age, and studies on sudden hearing losses and tinnitus
- Studies on micronutrients that are not vitamin or antioxidant
- Studies that have examined the effect of vitamin/antioxidant supplementation along with other agents
- Studies conducted on children or adolescents
- Studies on the effects of medications on improving patients with impaired hearing
- Protocol studies or studies with no report of results
- Studies that were not in English
- Inaccessible articles

### 2.3 Study selection

After the initial search of databases and removing the duplicated articles, screening of titles and abstracts was conducted by two independent researchers (BP and MA) to exclude irrelevant articles as well as those that did not meet the considered inclusion criteria. The full texts of the remaining related articles were then carefully evaluated by these two researchers (BP and MA) to select appropriate articles based on the methodology and results. Any inconsistency between the researchers was fixed by consulting with the third researcher (MOT).

### 2.4 Data extraction

Two independent researchers (MA and MOT) extracted the following information from selected studies: author's name, study location, study design, study population, mean age, gender, sample size, type, dose and duration of intervention (clinical trials), control group, serum vitamins/antioxidants (cross‐sectional and cohort studies), and outcomes.

### 2.5 Quality assessment

JBI critical appraisal checklist for cross‐sectional, cohort, and clinical trial studies was used to evaluate the quality of articles examined in our study.^33^ Criteria for each type of study are presented separately, for example, clinical trial criteria used included: (a) random sequence generation, (b) allocation concealment, (c) similarity of treatment groups at baseline, (d) blinding of participants, (e) blinding of those delivering treatment, (f) blinding of outcome assessors, (g) identical treatment of treatment groups except for the intervention of interest, (h) completing the follow‐up and explanation and analyzing the differences between the two groups in the field of follow‐up, (i) analyzing participants in randomly selected groups, (j) measuring results for treatment groups similarly, (k) outcome measurement in a valid and reliable way, (l) use appropriate statistical analysis, and (m) appropriateness of trial design and calculate deviation from the standard RCT design (individual randomization, parallel groups) in the conduct and analysis of the trial.

Three final conclusions were considered for quality assessment. So, according to the answers that were given to the criteria, each study was graded as follows: 1‐include (low risk of bias), 2‐exclude (high risk of bias), and 3‐seek for information (unclear risk of bias).

## 3 RESULTS

### 3.1 Study selection

**FIGURE 1:**
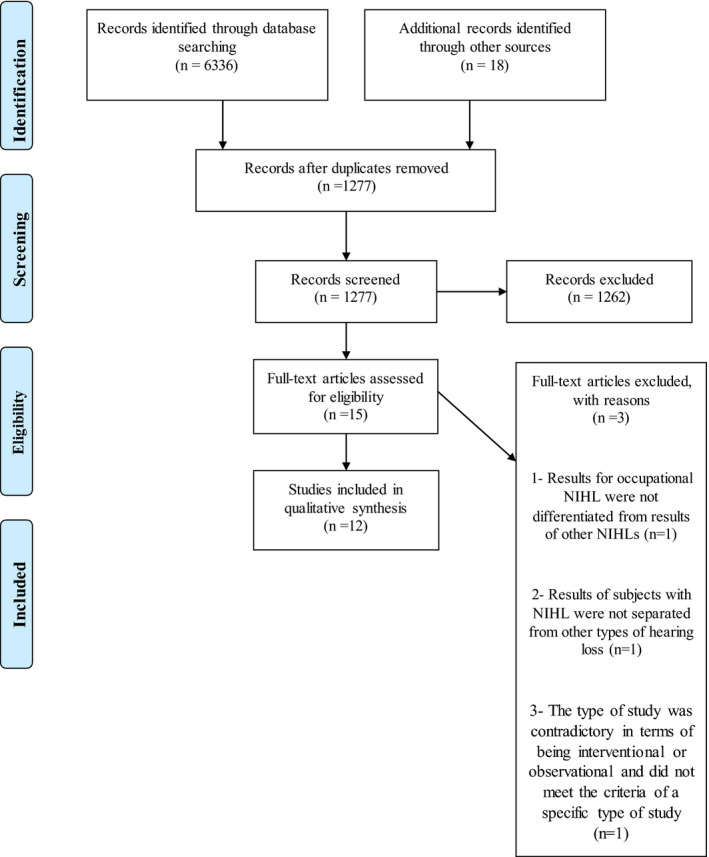
Flow chart of the study selection process

A flow chart depicting the study selection process is presented in Figure 1. Using the key terms of the study, the initial database search provided 6336 articles, and 18 additional articles were identified from other sources. First, duplicate articles (n = 5077) were removed and the titles and abstracts of another 1277 articles were screened and 1262 studies with exclusion criteria or without inclusion criteria were excluded. Then, the full texts of the remaining 15 articles were assessed from which three articles were excluded for the following reasons: (a) Results for occupational NIHL were not differentiated from results of other NIHLs (n = 1),^34^ (b) Results of subjects with NIHL were not separated from other types of hearing loss (n = 1),^35^ and (c) The type of study was contradictory in terms of being interventional or observational and did not meet the criteria of a specific type of study.^36^ Although 12 articles were finally found, one of them^37^ could be viewed as two separate trials, because it has examined the effects of two different antioxidants (ginseng and NAC) on NIHL, separately.

### 3.2 Study and participant characteristics

The characteristics of the studies included in the present systematic review are shown in Table 1. As noted, four studies have been conducted in USA,^38^, ^39^, ^40^, ^41^ one in Sweden,^42^ and one in each Turkey,^43^ Taiwan,^44^ India,^45^ Italy,^25^ Iran,^37^ Israel,^46^ and China.^47^ Among these studies, eight were clinical trials,^25^, ^37^, ^39^, ^40^, ^42^, ^44^, ^45^, ^47^ of which two studies were crossover in design.^39^, ^44^ Three studies were cross‐sectional,^38^, ^43^, ^46^ one study was cohort.^41^ Four studies included both males and females,^25^, ^38^, ^42^, ^47^ one study included only females,^41^ and other seven studies were performed merely on male adults.^37^, ^39^, ^40^, ^43^, ^44^, ^45^, ^46^ The age range of the participants was 23‐82 years old in these studies. The sample size varied noticeably between the studies, ranging from 20 to 566 subjects in clinical trials and 58 to 60 in cross‐sectional studies. In addition, the sample sizes were 12 789 people in cohort study. Of the 13 studies observed in Table 1, six of them examined vitamins,^38^, ^39^, ^41^, ^43^, ^45^, ^46^ four of them NAC,^37^, ^40^, ^42^, ^44^ one of them alpha‐lipoic acid,^25^ one ginseng,^37^ and one zinc gluconate.^47^

**TABLE 1:** Characteristics of the studies that were included in the systematic review

	Author (year) (reference)	Country	Study design	Population	Mean age	Gender	Sample size	Type, dose, and duration of intervention (clinical trial and observational studies)	Control group	Serum vitamins/antioxidants (cross‐sectional and cohort studies)	Outcomes
1	Rabinowitz et al (2002)^38^	USA	Cross‐sectional	Noise‐exposed workers	34.3	Male/female	Case: 58	‐	‐	Vit E Vit C	1‐Audiometric high (3, 4, 6 kHz) and low (0.5, 1, 2 kHz) frequency average 3‐High (F2 = 3, 3.5, 4, 4.5, 5 kHz) and low (F2 = 1.5, 2, 2.5 kHz) frequency OAE amplitude average
2	Shemesh et al (1993)^46^	Israel	Cross‐sectional	Army personnel with NIHL	39.4	Male	Case: 29 Control: 27	‐	‐	Vitamin B12	The hearing threshold changes
3	Gok et al (2004)^43^	Turkey	Cross‐sectional	Subjects with noise‐induced hearing loss	36.9	Male	Case: 28 Control: 32	‐	‐	Homocysteine, Folic acid, and Vitamin B12	The hearing threshold changes
4	Lin et al (2010)^44^	Taiwan	Prospective double‐blind, crossover design clinical trial	Male workers	82.1	Male	Case: 53 Control: 53	N‐Acetylcysteine (1200 mg/day), 14 d	Placebo (a tablet of identical taste and odor to the NAC agent)	‐	1‐The hearing threshold changes 2‐TTS in low‐ and high‐frequency
5	Le Prell et al (2011)^39^	USA	Double‐blind, crossover randomized clinical trial	Military personnel	25.4	Male	Case: 31 Control: 31	Combination of B‐carotene (18 mg), Vitamin C (500 mg), Vitamin E (305 mg), Magnesium (1949 mg) (6 pills daily)	Placebo (inactive tablets identical in appearance to the micronutrients pill)	‐	Changes in auditory function using conventional pure‐tone thresholds and DPOAE amplitudes as metrics
6	Lindblad et al (2011)^42^	Sweden	Clinical trial	Military officers in the Swedish Army	29	Male/female	Case: 11 Control: 23	NAC (200 mg). 4 tablets were taken after shooting session in a bunker‐like room: 1‐ Directly after exposure, 2‐ 1 h later, 3‐ At breakfast the next day and 4‐ An hour later	Placebo	‐	Tone thresholds, TEOAE with and without contralateral noise, PMTF and thresholds for brief tones in modulated noise
7	Kapoor et al (2011)^45^	India	Clinical trial	Industrial army Base workshop workers	36.4	Male	Case: 10 Control: 10	Vitamin E (400 mg/day) for 6 d	Subjects were only exposed to noise	‐	Mean temporary threshold shift of the combined left and right ear
8	Quaranta et al (2012)^25^	Italy	Randomized clinical trial	Young normally hearing subjects.	23.9	Male/female	Case: 10 Control: 10	Alpha‐lipoic acid (600 mg), 10 d before exposed to noise and 1 h before exposed to noise	Subjects were only exposed to noise	‐	1‐ Pre‐exposure thresholds at 3,4,6 kHz 2‐ TTS at 3,4,6 kHz 3‐ TEOAEs amplitude change after noise exposure
9	Doosti et al (2014)^37^	Iran	Randomized clinical trial	Textile workers	39.12	Male	Case: 19 Control: 19	NAC (1200 mg/once a day) for 14 days	Received no supplement.	‐	Noise‐induced Pure tone audiometry and high‐frequency audiometry
10	Doosti et al (2014)^37^	Iran	Randomized clinical trial	Textile workers	39.12	Male	Case: 19 Control: 19	Ginseng (200 mg/once a day) for 14 d	Received no supplement	‐	Pure tone audiometry and high‐frequency audiometry
11	Kopke et al (2015)^40^	USA	Prospective, randomized, double‐blind, controlled trial	Military population after weapons training	26.5	Male	Case: 277 Control: 289	NAC (2700 mg/day), for each of the first 13 d of weapons training	Placebo	‐	Primary outcomes 1‐Rate of subjects demonstrating STS, using pure tone 2‐Differences in frequency, intensity, or types of documented adverse events Secondary outcomes: 1‐Pure tone threshold 2‐Rate of subjects demonstrating STS, using pure tone trigger hand ear
12	Yeh et al (2019)^47^	China	Clinical trial	Subjects with tinnitus and NIHL	47.7	Male/Female	Case: 20 Control: 20	Zinc gluconate (40 mg/day) for 2 mo	No placebo	‐	1‐Mandarin‐Chinese version of THI questionnaire 2‐DPOAE 3‐Tinnitus loudness 4‐Tinnitus frequency (Hz) 5‐Hearing threshold
13	Curhan et al (2015)^41^	United States	Prospective cohort study from 1991 to 2009	Cases of incident hearing loss	36.3	Female	65 521	‐	‐	Carotenoid, Vitamin A, Vitamin C, Vitamin E, and Folate intake	Risk of hearing loss bases on RR

Note

‐, Not applicable.

Abbreviations: DPOAE, distortion product otoacoustic emission; Hz, hertz; kHz, kilohertz; NAC, N‐Acetylcysteine;OAE, otoacoustic emissions; PMTF, psychoacoustical modulation transfer function; RR, risk ratio; STS, Standard Threshold Shift; TEOAE, Transient Evoked Otoacoustic Emissions; THI, Tinnitus Handicap inventory; TTS, temporary threshold shift.

### 3.3 Quality assessment

The quality of the studies was independently assessed by two researchers (BP and MA) according to the “Critical Appraisal tools for use in JBI Systematic Reviews” and all studies appeared to have fair to good quality (low risk of bias), so they were included in the systematic review (Appendix A).

### 3.4 Main findings

#### 3.4.1 Evidence from cross‐sectional studies

In 1993, Shemesh et al^46^ studied the relationship between vitamin B12 deficiency and NIHL. The results of this study showed that incidence of vitamin B12 deficiency among subjects with NIHL was greater than subjects who had normal audiogram and it was 27% and 19%, respectively.^46^ Gok et al^43^ also confirmed these findings in their study, which revealed that serum vitamin B12 and folic acid levels in subjects with NIHL were significantly lower than subjects without NIHL. Indeed, the results of this study showed that mean ± standard deviation (SD) of vitamin B12 and folic acid levels in NIHL group were 199.87 ± 75.25 pmol/L and 10.71 ± 4.16 nmol/L, respectively, while they were 323.62 ± 121.92 pmol/L and 12.69 ± 3.61 nmol/L, respectively, in the control group.^43^ On the other hand, Rabinowitz et al^38^ studied the role of vitamin E and vitamin C on hearing status of 58 industrial workers and found that vitamin C had no significant association with either audiometry or otoacoustic emissions (OAEs) but there was a non‐protective correlation between serum levels of vitamin E and hearing function. They stated that high‐frequency audiometric results got worse in higher levels of vitamin E but this relation was not strong in high‐frequency OAEs.^38^

#### 3.4.2 Evidence from clinical trial studies

Kapoor et al^45^ conducted a study among a group of 40 male industrial workers and found that consuming vitamin E (400 milligram [mg]/day) for six consecutive working days can protect hearing system against noise exposure especially at 0.25, 0.5, and 1.0 kilohertz (kHz). Perll et al^39^ in their clinical trial delivered a set of supplementations including b‐carotene (18 mg), vitamin C (500 mg), vitamin E (305 mg), and magnesium (1949 mg) as full daily dose (three pills twice daily) to 31 subjects to examine whether these supplementations can prevent NIHL or not. The results of this study showed that simultaneous consumption of the aforementioned had no significant effect on NIHL.^39^ On the other hand, Lin et al^44^ studied the protective effects of N‐acetylcysteine (NAC) (1200 mg/day, 14 days) on hearing loss in male workers. Workers were given NAC for 2 weeks and after 2 weeks of washout period, they were given a placebo for another 2 weeks. This sequence was reversed for some of the workers who were randomly selected. The results indicated that noise‐induced TTS at high frequency after placebo and after NAC was 2.8 decibels (dB) and 2.5 dB, respectively, which were significantly different (*P*‐value: .03), but at low frequency, this difference in noise‐induced TTS between the post‐NAC (1.2 dB) and post‐placebo (0.9 dB) was not significant (*P*‐value: .88).^44^ Also, Lindblad and colleagues showed in a prospective study that consuming 200 mg NAC after acute noise exposure could reduce the threshold variability in the left ear.^42^ The most significant finding of this study was that the non‐linearity of the cochlea that was strongly decreased in the control group, as revealed by the psychoacoustical modulation transfer function (PMTF) results, was practically unchanged in the NAC group throughout the trial. Moreover, NAC expedited the recovery after temporary hearing loss in this study.^42^ In the same way, Doosti and colleagues showed that workers who received 1200 mg/day of NAC for 14 days experienced less TTS at 4, 8, and 16 KHz (*P*, .001) in both ears.^37^ They observed similar results for taking 200 mg/day of ginseng as an antioxidant; however, the protective effect of NAC was greater.^37^ Also, in a prospective clinical trial, efficacy of NAC in prevention of NIHL was investigated by Kopke and colleagues.^40^ In their study, subjects were administered 2700 mg of NAC for 13 consecutive days of weapons training and the results indicated that there were no significant differences for the primary and secondary outcomes but standard threshold shift (STS) rate in the trigger hand ear did show a significant difference (34.98% for placebo, 27.14% for NAC, and *P*‐value ¼ .0288).^40^

Alpha‐lipoic acid (ALA) as a powerful lipophilic antioxidant can protect hearing against noise exposure. Quaranta et al^25^ evaluated the effect of ALA on temporary hearing loss and found that consuming 600 mg ALA for 10 consecutive days can significantly protect from temporary hearing loss induced by exposure to 90 dB pure tone of high frequency, and that TTS and Transient Evoked Otoacoustic Emissions (TEOAEs) amplitude change after noise exposure were lower after 10 days of oral ingestion of ALA in comparison to 1 hour after ingestion.^25^ Finally, in a study by Yeh et al,^47^ subjects with NIHL‐associated tinnitus took 40 mg/day zinc gluconate (Zinga 78 mg and 10 mg elemental zinc) for 2 months. The results demonstrated that there were no statistically significant differences in hearing thresholds, speech reception thresholds, or tinnitus frequency and loudness results before and after treatment, but following zinc treatment, Tinnitus Handicap inventory (THI) scores improved significantly in patients with NIHL‐associated tinnitus.^47^

#### 3.4.3 Evidence from cohort studies

In a prospective cohort study conducted by Curhan et al,^41^ 65 521 females in the Nurses' Health Study II were observed from 1991 to 2009 to investigate the relationship between consumption of vitamin A, vitamin C, vitamin E, carotenoids, and folate and risk of hearing loss. The results of this study indicated that there was no significant relationship between intake of vitamin E and the risk of hearing loss, while higher intake of vitamin C (≥1000 mg/day) was related to higher risk of hearing loss in comparison to lower intake of this vitamin (<75 mg/day). Also, higher intakes of b‐cryptoxanthin and b‐carotene were related to lower risk of hearing loss. No significant relationship was observed for intakes of other carotenoids or vitamin A. In the case of folate, they observed that lower intake of folate (<200 microgram [µg]/day) was associated with higher risk of hearing loss in comparison to 200‐399 µg/day.^41^

### 3.5 Meta‐analysis

It was not possible to perform a meta‐analysis on the studies, because of the high heterogeneity of the studies in addition to inconsistent exposure and outcome measures.

## 4 DISCUSSION

A review of the protective effects of vitamins/antioxidants on occupational NIHL has been conducted, and this is the first study that systematically review this topic. Based on our search results, 12 articles with 13 arms were qualified to be included in the current study. According to the review of the qualified studies, it can be acknowledged that vitamin B12, folic acid, and NAC have a significant protective effect on NIHL, but the protective effect of vitamins E, C, and A on NIHL is unproven.

So far, some review studies have examined the association between hearing loss and vitamins/antioxidants. For example, a review study by da costa et al on the effect of supplementation with antioxidants on auditory threshold in sensorineural hearing loss showed that ginseng prevented auditory threshold worsening in the 4‐kHz, but not at the 6‐kHz frequency in patients with sensorineural hearing loss caused by exposure to high levels of sound pressure. However, there was no enhancement in the thresholds with vitamin E supplementation.^28^ Also, Jung et al, in their review study in the field of association between nutritional factors with hearing loss, found that various nutritional factors (such as vitamins A, C, and E, and zinc) are associated with hearing status, and the incidence of hearing loss was increased with the lack of these micronutrients.^30^ Alvarado et al in their study showed that combination of some or all of the antioxidants, such as NAC, vitamins A, C, and E, and magnesium (Mg), can affect on their mechanisms of action, potentiating the optimistic effect over noise overexposure.^31^

Elderly subjects are more vulnerable to NIHL than younger individuals, and factors independently but causally associated to age are important in the development of NIHL among workers exposed to noise.^48^ A scoping review study on age‐related hearing loss (ARHL) by Rodrigo et al showed that the vitamin C supplementation significantly decreases the permanent hearing threshold in the medium frequency range, while its deficiency has no effect on hearing loss. Also, the consumption of beta‐carotene, vitamins C and E, as well as Mg increases the average PTA response at high frequencies, especially in the combination form, but no significant association was seen between serum vitamin B12 with hearing loss. On the other hand, it was observed that people with moderate levels of folic acid have 32% lower odds of experiencing hearing loss at lower frequencies.^29^ Also, the cohort study by Gopinath et al reported that the high level of consumption of vitamins A and E was inversely associated with the incidence of ARHL, but 5‐year longitudinal analysis did not show any relationship with the incidence of ARHL.^49^ So, despite the differences in etiology and pathophysiology of NIHL and ARHL, it can be stated that some applied interventions for ARHL can be feasible for NIHL, such as folic acid, because they have some overlaps as well.

Vitamin E efficiently neutralizes free radicals in reduced oxygen. It also prevents glutamate release and then prevents glutamate‐induced neurotoxicity.^27^ But researchers have reported contradictory results regarding protective effects of vitamin E against NIHL. Kapoor et al reported that vitamin E protects against the adverse effect of noise on hearing at frequencies 0.25, 0.5, and 1.0 kHz.^45^ Conversely, Gök et al stated that there was no association between vitamin E and hearing loss.^50^ Based on the obtained results, it is concluded that vitamin E can provide a protective effect against low‐frequency noise^45^ but not against high‐frequency noise.^38^ This result has also been found for vitamin C. Vitamin C is a water‐soluble antioxidant vitamin and is principal to preserve vitamin E levels by recycling the oxidized form of vitamin E to the reduced form.^27^ In animal models, it is proven that vitamin C can provide a protective effect against hearing loss,^51^, ^52^ but studies conducted in the human cases related to non‐occupational hearing problems have shown conflicting results,^49^, ^53^ and in our reviewed studies, no effect was observed between vitamin C and decreased hearing loss.^38^, ^39^, ^41^ Several reasons may justify these differences, which can be motivation for designing longitudinal cohort study.

The protective effect of B12 and folic acid on NIHL has also been studied. As mentioned above, documents have proved that B12 and folic acid can provide a protective effect for NIHL up to now. Vitamin B12 and folic acid are effective on homocysteine metabolism and their deficiency increases homocysteine levels. Increased homocysteine levels may cause reductions in intracellular concentrations of glutathione, which increased lipid peroxidation. Also hyperhomocysteinemia has been associated with elevations in tissue iron stores and increased in vivo lipid peroxidation. Homocysteine might also render neurons vulnerable to excitotoxicity by inducing DNA damage and impaired transmethylation of DNA.^53^ On the other hand, Shemesh et al^46^ have shown that incidence of vitamin B12 (cobalamin) deficiency among subjects with NIHL is greater than with none‐NIHL subjects. Moreover, it is confirmed that serum vitamin B12 and folic acid levels in subjects with NIHL are significantly lower than subjects without NIHL.^43^ In another study, it was found that low serum levels of folic acid are significantly related to high‐frequency hearing loss.^54^ The results have further shown that cobalamin deficiency is strongly associated with hearing loss at high frequencies.^54^ Other studies have also confirmed that folate and cobalamin have a significant relationship with auditory functions.^43^, ^46^, ^50^, ^54^ Mechanisms and metabolic roles of cobalamin and folic acid are interrelated and they reduce with increasing age, so their effects may be controversial. In the case of effects of vitamin A, the results of the study by Curhan et al^41^ have indicated that b‐cryptoxanthin and b‐carotene are related to lower risk of hearing loss with no significant relationship between intakes of other carotenoids or vitamin A and hearing loss. Vitamin A family (eg, carotenoids, vitamin A, etc) can provide vascular or antioxidative benefits and the retinoic acid pathway has been identified as a promising target for the development of prevention and treatment strategies for sensorineural hearing loss. Also in animal models, b‐carotene and vitamins A have been shown to have protective effects against hearing loss.^41^ Cohort study mentioned above was a study of 65 521 women with repeated dietary assessments and long‐term follow‐up from 1991 to 2009; therefore, it can be reliable in terms of sample size and follow‐up time. However, more studies are needed for definitive conclusions.

NAC is one of the first nutrient‐based antioxidants brought to clinical trials and has been most thoroughly investigated in NIHL protection. NAC is known as Mucomyst. It is a free radical scavenger and can increase glutathione (GSH) production, which has an important role in limiting noise‐induced cochlear damage through reacting directly with oxidants (ROS), and inhibiting oxidation of the molecules.^37^ The results of study by Lin et al^44^ in male workers have indicated that mean hearing loss at high frequency after placebo and after NAC is significantly different, but not at low frequency.^44^ The researchers have perceived that NAC could also be effective in reducing acute hearing loss. In this regard, Lindblad et al^42^ have shown that consuming NAC after acute noise exposure could reduce the adverse effect of noise exposure on the hearing threshold. This protective effect has also been investigated and confirmed at frequencies of 4, 8, and 16 kHz.^37^ It has been suggested that this effect results from the free radical scavenger characteristic of NAC. A similar result has been observed for ginseng as an antioxidant, though the protective effect of NAC has been greater.^37^ The protective effect of NAC is not only against NIHL due to exposure to continuous noise but also there is some evidence that it can provide protection against acoustic accidents like shooting, although results are somewhat controversial.

Among other antioxidants that were investigated for their protection against noise exposure, ALA and zinc gluconate have been considered more significantly.^25^, ^47^ ALA is an important cofactor in mitochondrial enzymes. It is a new biological antioxidant and a powerful free radical scavenger, and has been shown in animal models to protect against hearing loss due to age and cisplatin.^25^ Zinc also plays a substantial antioxidant role in the human metabolism. The results of studies on these supplements are opposite to one another; ALA could significantly protect from temporary hearing loss induced by exposure to 90 dB pure ton at high frequency (6 kHz), while there was no significant protection reported for zinc gluconate. Due to the small number of studies conducted in this field, more studies are needed for a conclusive result to these two factors.

The current systematic review has some strengths, such as, according to the search results, this is the first study that systematically reviewed the protective effects of vitamins/antioxidants on occupational NIHL. The results of this study are economically important for industries because they can prevent hearing loss by recommending to receive B12, folic acid, and NAC as antioxidants from various food sources or supplements at much lower costs than the cost of administrative and engineering. Also, there are a number of limitations to this article, including it was not registered in PROSPERO, due to the delay in receiving registration code, caused by COVID‐19 epidemic conditions. The articles reviewed in this study were either observational or interventional, due to the small number of articles worked in this field, which is another limitation itself. On the other hand, this has led to researchers not being able to review only one specific exposure and outcome. Also, it was not possible to perform a meta‐analysis on the studies, because of the high heterogeneity among the studies.

## 5 CONCLUSION

In general, based on the literature review, it can be concluded that vitamin B12, folic acid, and NAC have a significant protective effect on NIHL. However, the protective effects of these antioxidants against hearing loss at different frequencies are not yet known. Findings regarding the protective effect of vitamins E, C, and A on NIHL are inconsistent. Therefore, in order to achieve conclusive results, more interventional and cohort studies are needed.

## DISCLOSURE

*Approval of the research protocol*: NA. *Informed consent*: NA. *Registry and the registration no. of the study/trial*: It was not registered in PROSPERO. *Animal studies*: NA. *Conflict of interest*: The authors declare that they have no competing interests.

## AUTHOR CONTRIBUTIONS

MA and BP conceived the study and designed the search strategy; BP and MA conducted the study selection; MA and MOT conducted data extraction; BP and MA evaluated the quality of bias of included studies; B.P and MA and MOT wrote the first draft of the manuscript. All authors read and approved the final version of the manuscript.

## Supporting information

Supplementary Material

## Data Availability

All data and materials are available in full in manuscript, figure, and tables.
