# Supplementary material for: Protective effects of vitamins/antioxidants on occupational noise‐induced hearing loss: A systematic review
Source: J Occup Health. 2021 Mar 31;63(1):e12217. doi: 10.1002/1348-9585.12217 (PMC8011460; doi:10.1002/1348-9585.12217)
Supplement: Supplementary file 1 — Supplementary Material [file JOH2-63-e12217-s001.docx]

**Appendix A:** BI critical appraisal checklist for cross-sectional, cohort and clinical trial studies

**1- Author:** Gok, U (2004)

| Yes | | No | Unclear | Not applicable |
| --- | --- | --- | --- | --- |
| 1. Were the criteria for inclusion in the sample clearly defined? |  | □ | □ | □ |
| 2. Were the study subjects and the setting described in detail? |  | □ | □ | □ |
| 3. Was the exposure measured in a valid and reliable way? | □ | □ | □ |  |
| 4. Were objective, standard criteria used for measurement of the condition? |  | □ | □ | □ |
| 5. Were confounding factors identified? | □ | □ |  | □ |
| 6. Were strategies to deal with confounding factors stated? | □ | □ |  | □ |
| 7. Were the outcomes measured in a valid and reliable way? |  | □ | □ | □ |
| 8. Was appropriate statistical analysis used? |  | □ | □ | □ |

**Overall appraisal:**

Include Exclude Seek further info

Comments (Including reason for exclusion)

**2-Author:** Rabinowitz, P. M (2002)

| Yes | | No | Unclear | Not applicable |
| --- | --- | --- | --- | --- |
| 1. Were the criteria for inclusion in the sample clearly defined? | □ | □ |  | □ |
| 2. Were the study subjects and the setting described in detail? |  | □ | □ | □ |
| 3. Was the exposure measured in a valid and reliable way? |  | □ | □ | □ |
| 4. Were objective, standard criteria used for measurement of the condition? |  | □ | □ | □ |
| 5. Were confounding factors identified? |  | □ | □ | □ |
| 6. Were strategies to deal with confounding factors stated? |  | □ | □ | □ |
| 7. Were the outcomes measured in a valid and reliable way? |  | □ | □ | □ |
| 8. Was appropriate statistical analysis used? |  | □ | □ | □ |

**Overall appraisal:**

Include Exclude Seek further info

Comments (Including reason for exclusion)

**3-Author:** Shemesh, Z (1993)

| Yes | | No | Unclear | Not applicable |
| --- | --- | --- | --- | --- |
| 1. Were the criteria for inclusion in the sample clearly defined? | □ | □ |  | □ |
| 2. Were the study subjects and the setting described in detail? |  | □ | □ | □ |
| 3. Was the exposure measured in a valid and reliable way? | □ | □ | □ |  |
| 4. Were objective, standard criteria used for measurement of the condition? |  | □ | □ | □ |
| 5. Were confounding factors identified? |  | □ | □ | □ |
| 6. Were strategies to deal with confounding factors stated? | □ | □ |  | □ |
| 7. Were the outcomes measured in a valid and reliable way? |  | □ | □ | □ |
| 8. Was appropriate statistical analysis used? |  | □ | □ | □ |

**Overall appraisal:**

Include Exclude Seek further info

Comments (Including reason for exclusion)

**JBI CRITICAL APPRAISAL CHECKLIST FOR COHORT STUDIES**

**1-Author:** Curhan, S. G (2015)

| Yes | | No | Unclear | Not applicable |
| --- | --- | --- | --- | --- |
| 1. Were the two groups similar and recruited from the same population? | □ | □ | □ |  |
| 2. Were the exposures measured similarly to assign people  to both exposed and unexposed groups? | □ | □ | □ |  |
| 3. Was the exposure measured in a valid and reliable way? |  | □ | □ | □ |
| 4. Were confounding factors identified? |  | □ | □ | □ |
| 5. Were strategies to deal with confounding factors stated? |  | □ | □ | □ |
| 6. Were the groups/participants free of the outcome at the start of the study (or at the moment of exposure)? | □ | □ | □ |  |
| 7. Were the outcomes measured in a valid and reliable way? | □ | □ |  | □ |
| 8. Was the follow up time reported and sufficient to be long enough for outcomes to occur? |  | □ | □ | □ |
| 9. Was follow up complete, and if not, were the reasons to loss to follow up described and explored? |  | □ | □ | □ |
| 10. Were strategies to address incomplete follow up utilized? | □ | □ | □ |  |
| 11. Was appropriate statistical analysis used? |  | □ | □ | □ |

**Overall appraisal:**

Include Exclude Seek further info

Comments (Including reason for exclusion)

**JBI CRITICAL APPRAISAL CHECKLIST FOR RANDOMIZED CONTROLLED TRIALS**

**1-Author:** Lin, C. Y (2010)

| Yes | | No | Unclear | NA |
| --- | --- | --- | --- | --- |
| 1. Was true randomization used for assignment of participants to treatment groups? | □ | □ |  | □ |
| 2. Was allocation to treatment groups concealed? | □ | □ |  | □ |
| 3. Were treatment groups similar at the baseline? |  | □ | □ | □ |
| 4. Were participants blind to treatment assignment? |  | □ | □ | □ |
| 5. Were those delivering treatment blind to treatment assignment? | □ | □ |  | □ |
| 6. Were outcomes assessors blind to treatment assignment? | □ | □ |  | □ |
| 7. Were treatment groups treated identically other than the intervention of interest? |  | □ | □ | □ |
| 8. Was follow up complete and if not, were differences between groups in terms of their follow up adequately described and analyzed? |  | □ | □ | □ |
| 9. Were participants analyzed in the groups to which they were randomized? | □ | □ | □ |  |
| 10. Were outcomes measured in the same way for treatment groups? |  | □ | □ | □ |
| 11. Were outcomes measured in a reliable way? |  | □ | □ | □ |
| 12. Was appropriate statistical analysis used? |  | □ | □ | □ |
| 13. Was the trial design appropriate, and any deviations from the standard RCT design (individual randomization, parallel groups) accounted for in the conduct and analysis of the trial? |  | □ | □ | □ |

**Overall appraisal:**

Include Exclude Seek further info

Comments (Including reason for exclusion)

2- **Author:** Le Prell, C. G (2011)

| Yes | | No | Unclear | NA |
| --- | --- | --- | --- | --- |
| 1. Was true randomization used for assignment of participants to treatment groups? | □ | □ |  | □ |
| 2. Was allocation to treatment groups concealed? | □ | □ |  | □ |
| 3. Were treatment groups similar at the baseline? |  | □ | □ | □ |
| 4. Were participants blind to treatment assignment? |  | □ | □ | □ |
| 5. Were those delivering treatment blind to treatment assignment? | □ | □ |  | □ |
| 6. Were outcomes assessors blind to treatment assignment? | □ | □ |  | □ |
| 7. Were treatment groups treated identically other than the intervention of interest? |  | □ | □ | □ |
| 8. Was follow up complete and if not, were differences between groups in terms of their follow up adequately described and analyzed? |  | □ | □ | □ |
| 9. Were participants analyzed in the groups to which they were randomized? | □ | □ | □ |  |
| 10. Were outcomes measured in the same way for treatment groups? |  | □ | □ | □ |
| 11. Were outcomes measured in a reliable way? |  | □ | □ | □ |
| 12. Was appropriate statistical analysis used? |  | □ | □ | □ |
| 13. Was the trial design appropriate, and any deviations from the standard RCT design (individual randomization, parallel groups) accounted for in the conduct and analysis of the trial? |  | □ | □ | □ |

**Overall appraisal:**

Include Exclude Seek further info

Comments (Including reason for exclusion)

3- **Author:** Lindblad, A. C (2011)

| Yes | | No | Unclear | NA |
| --- | --- | --- | --- | --- |
| 1. Was true randomization used for assignment of participants to treatment groups? | □ | □ |  | □ |
| 2. Was allocation to treatment groups concealed? | □ | □ |  | □ |
| 3. Were treatment groups similar at the baseline? | □ | □ |  | □ |
| 4. Were participants blind to treatment assignment? | □ | □ |  | □ |
| 5. Were those delivering treatment blind to treatment assignment? | □ | □ |  | □ |
| 6. Were outcomes assessors blind to treatment assignment? | □ | □ |  | □ |
| 7. Were treatment groups treated identically other than the intervention of interest? | □ | □ |  | □ |
| 8. Was follow up complete and if not, were differences between groups in terms of their follow up adequately described and analyzed? | □ | □ |  | □ |
| 9. Were participants analyzed in the groups to which they were randomized? | □ | □ |  | □ |
| 10. Were outcomes measured in the same way for treatment groups? |  | □ | □ | □ |
| 11. Were outcomes measured in a reliable way? |  | □ | □ | □ |
| 12. Was appropriate statistical analysis used? |  | □ | □ | □ |
| 13. Was the trial design appropriate, and any deviations from the standard RCT design (individual randomization, parallel groups) accounted for in the conduct and analysis of the trial? | □ | □ |  | □ |

**Overall appraisal:**

Include Exclude Seek further info

Comments (Including reason for exclusion)

4- **Author:** Kapoor, N. (2011)

| Yes | | No | Unclear | NA |
| --- | --- | --- | --- | --- |
| 1. Was true randomization used for assignment of participants to treatment groups? | □ | □ |  | □ |
| 2. Was allocation to treatment groups concealed? | □ | □ |  | □ |
| 3. Were treatment groups similar at the baseline? |  | □ | □ | □ |
| 4. Were participants blind to treatment assignment? | □ | □ |  | □ |
| 5. Were those delivering treatment blind to treatment assignment? | □ | □ |  | □ |
| 6. Were outcomes assessors blind to treatment assignment? | □ | □ |  | □ |
| 7. Were treatment groups treated identically other than the intervention of interest? | □ |  | □ | □ |
| 8. Was follow up complete and if not, were differences between groups in terms of their follow up adequately described and analyzed? | □ | □ |  | □ |
| 9. Were participants analyzed in the groups to which they were randomized? | □ | □ |  | □ |
| 10. Were outcomes measured in the same way for treatment groups? |  | □ | □ | □ |
| 11. Were outcomes measured in a reliable way? |  | □ | □ | □ |
| 12. Was appropriate statistical analysis used? |  | □ | □ | □ |
| 13. Was the trial design appropriate, and any deviations from the standard RCT design (individual randomization, parallel groups) accounted for in the conduct and analysis of the trial? | □ | □ |  | □ |

**Overall appraisal:**

Include Exclude Seek further info

Comments (Including reason for exclusion)

5- **Author:** Quaranta, N (2012)

| Yes | | No | Unclear | NA |
| --- | --- | --- | --- | --- |
| 1. Was true randomization used for assignment of participants to treatment groups? | □ | □ |  | □ |
| 2. Was allocation to treatment groups concealed? | □ | □ |  | □ |
| 3. Were treatment groups similar at the baseline? |  | □ | □ | □ |
| 4. Were participants blind to treatment assignment? | □ | □ |  | □ |
| 5. Were those delivering treatment blind to treatment assignment? | □ | □ |  | □ |
| 6. Were outcomes assessors blind to treatment assignment? | □ | □ |  | □ |
| 7. Were treatment groups treated identically other than the intervention of interest? | □ |  | □ | □ |
| 8. Was follow up complete and if not, were differences between groups in terms of their follow up adequately described and analyzed? | □ | □ |  | □ |
| 9. Were participants analyzed in the groups to which they were randomized? | □ | □ |  | □ |
| 10. Were outcomes measured in the same way for treatment groups? |  | □ | □ | □ |
| 11. Were outcomes measured in a reliable way? |  | □ | □ | □ |
| 12. Was appropriate statistical analysis used? |  | □ | □ | □ |
| 13. Was the trial design appropriate, and any deviations from the standard RCT design (individual randomization, parallel groups) accounted for in the conduct and analysis of the trial? | □ | □ |  | □ |

**Overall appraisal:**

Include Exclude Seek further info

Comments (Including reason for exclusion)

6- **Author:** Doosti, A (2014)

| Yes | | No | Unclear | NA |
| --- | --- | --- | --- | --- |
| 1. Was true randomization used for assignment of participants to treatment groups? |  | □ | □ | □ |
| 2. Was allocation to treatment groups concealed? | □ | □ |  | □ |
| 3. Were treatment groups similar at the baseline? |  | □ | □ | □ |
| 4. Were participants blind to treatment assignment? | □ | □ |  | □ |
| 5. Were those delivering treatment blind to treatment assignment? | □ | □ |  | □ |
| 6. Were outcomes assessors blind to treatment assignment? |  | □ | □ | □ |
| 7. Were treatment groups treated identically other than the intervention of interest? | □ |  | □ | □ |
| 8. Was follow up complete and if not, were differences between groups in terms of their follow up adequately described and analyzed? | □ | □ |  | □ |
| 9. Were participants analyzed in the groups to which they were randomized? | □ | □ |  | □ |
| 10. Were outcomes measured in the same way for treatment groups? |  | □ | □ | □ |
| 11. Were outcomes measured in a reliable way? |  | □ | □ | □ |
| 12. Was appropriate statistical analysis used? |  | □ | □ | □ |
| 13. Was the trial design appropriate, and any deviations from the standard RCT design (individual randomization, parallel groups) accounted for in the conduct and analysis of the trial? | □ | □ |  | □ |

**Overall appraisal:**

Include Exclude Seek further info

Comments (Including reason for exclusion)

7- **Author:** Kopke, R (2015)

| Yes | | No | Unclear | NA |
| --- | --- | --- | --- | --- |
| 1. Was true randomization used for assignment of participants to treatment groups? |  | □ | □ | □ |
| 2. Was allocation to treatment groups concealed? | □ | □ |  | □ |
| 3. Were treatment groups similar at the baseline? |  | □ | □ | □ |
| 4. Were participants blind to treatment assignment? |  | □ | □ | □ |
| 5. Were those delivering treatment blind to treatment assignment? |  | □ | □ | □ |
| 6. Were outcomes assessors blind to treatment assignment? |  | □ | □ | □ |
| 7. Were treatment groups treated identically other than the intervention of interest? |  | □ | □ | □ |
| 8. Was follow up complete and if not, were differences between groups in terms of their follow up adequately described and analyzed? | □ | □ |  | □ |
| 9. Were participants analyzed in the groups to which they were randomized? | □ | □ |  | □ |
| 10. Were outcomes measured in the same way for treatment groups? |  | □ | □ | □ |
| 11. Were outcomes measured in a reliable way? |  | □ | □ | □ |
| 12. Was appropriate statistical analysis used? |  | □ | □ | □ |
| 13. Was the trial design appropriate, and any deviations from the standard RCT design (individual randomization, parallel groups) accounted for in the conduct and analysis of the trial? |  | □ | □ | □ |

**Overall appraisal:**

Include Exclude Seek further info

Comments (Including reason for exclusion)

8- **Author:** Yeh, C. W (2019)

| Yes | | No | Unclear | NA |
| --- | --- | --- | --- | --- |
| 1. Was true randomization used for assignment of participants to treatment groups? | □ | □ |  | □ |
| 2. Was allocation to treatment groups concealed? | □ | □ |  | □ |
| 3. Were treatment groups similar at the baseline? |  | □ | □ | □ |
| 4. Were participants blind to treatment assignment? | □ | □ |  | □ |
| 5. Were those delivering treatment blind to treatment assignment? | □ | □ |  | □ |
| 6. Were outcomes assessors blind to treatment assignment? | □ | □ |  | □ |
| 7. Were treatment groups treated identically other than the intervention of interest? | □ |  | □ | □ |
| 8. Was follow up complete and if not, were differences between groups in terms of their follow up adequately described and analyzed? |  | □ | □ | □ |
| 9. Were participants analyzed in the groups to which they were randomized? | □ | □ | □ |  |
| 10. Were outcomes measured in the same way for treatment groups? |  | □ | □ | □ |
| 11. Were outcomes measured in a reliable way? |  | □ | □ | □ |
| 12. Was appropriate statistical analysis used? |  | □ | □ | □ |
| 13. Was the trial design appropriate, and any deviations from the standard RCT design (individual randomization, parallel groups) accounted for in the conduct and analysis of the trial? | □ | □ |  | □ |

**Overall appraisal:**

Include Exclude Seek further info

Comments (Including reason for exclusion)
